# Review of prostate cancer genomic studies in Africa

**DOI:** 10.3389/fgene.2022.911101

**Published:** 2022-10-11

**Authors:** Chaimae Samtal, Islam El Jaddaoui, Salsabil Hamdi, Laila Bouguenouch, Karim Ouldim, Chakib Nejjari, Hassan Ghazal, Hicham Bekkari

**Affiliations:** ^1^ Laboratory of Biotechnology, Environment, Agri-food and Health, Faculty of Sciences Dhar El Mahraz–Sidi Mohammed Ben Abdellah University, Fez, Morocco; ^2^ Laboratory of Human Pathologies Biology, Department of Biology, Faculty of Sciences, and Genomic Center of Human Pathologies, Faculty of Medicine and Pharmacy, University Mohammed V, Rabat, Morocco; ^3^ Laboratory of Environmental Health, Institut Pasteur Maroc, Casablanca, Morocco; ^4^ Faculty of Medicine, Pharmacy and Dentistry‒Sidi Mohammed Ben Abdellah University, University Hospital Hassan II, Fez, Morocco; ^5^ Department of Medicine, School of Medicine, Mohammed VI University of Health Sciences, Casablanca, Morocco; ^6^ School of Medicine and Pharmacy, Fes, Morocco; ^7^ Laboratory of Genomics and Bioinformatics, School of Pharmacy, Mohammed VI University of Health Sciences, Casablanca, Morocco; ^8^ National Center for Scientific and Technical Research, Rabat, Morocco

**Keywords:** prostate cancer, omics, genomics, variants, GWAS, Africa

## Abstract

Prostate cancer (PCa) is the second most commonly diagnosed in men worldwide and one of the most frequent cancers in men in Africa. The heterogeneity of this cancer fosters the need to identify potential genetic risk factors/biomarkers. Omics variations may significantly contribute to early diagnosis and personalized treatment. However, there are few genomic studies of this disease in African populations. This review sheds light on the status of genomics research on PCa in Africa and outlines the common variants identified thus far. The allele frequencies of the most significant SNPs in Afro-native, Afro-descendants, and European populations were compared. We advocate how these few but promising data will aid in understanding, better diagnosing, and precisely treating this cancer and the need for further collaborative research on the genomics of PCa in the African continent.

## Introduction

Prostate cancer (PCa) is currently the third most aggressive neoplasm globally. PCa has the second highest incidence woldwide in men (Jemal et al., 2008). PCa is the second most common malignancy diagnosed (after lung cancer) in men and the fifth leading cause of cancer death globally (Pernar et al., 2018). In 2018, an estimated 1,276,106 new cases of PCa were reported worldwide based on GLOBOCAN (Rawla, 2019). In 2020, according to the World Health Organization (WHO), 1,414,259 new cases were recorded, which is 7.3% of the total number of new cancer cases recorded (Giona et al., 2021). PCa is particularly widespread in developed countries. In the United States, 180,890 new cases were reported in 2016, with 26,120 deaths (Pernar et al., 2018). The PCa mortality rates are observed to be different among populations. The lowest mortality rates are in Asia, particularly in Eastern and South-Central Asia (Pernar et al., 2018). However, the highest rates are among populations in the Caribbean and Middle and Southern Africa (Pernar et al., 2018). The mortality rate of this disease increases with age, and almost 55% of all deaths occur after 65 years (Rawla, 2019). Compared to White men, African Americans have the highest incidence rates and develop aggressive variants of PCa, and their mortality is approximately twice that of White men. Social, environmental, and mainly genetic differences are integrated into this disparity (Rawla, 2019).

In Africa, the world’s second-largest and most populated continent, PCa is the most frequent cancer in men (Rebbeck, 2020). Numerous publications show that in men of African descent, PCa is considered the leading cancer in terms of incidence and mortality (Giona et al., 2021). The cancer incidences and fatality rates are different in the four African regions; however, it has been noticed that the incidence rate is lower in Northern Africa than in South, East, and West Africa. In Northern Africa (from 2002 to 2018), the incidence rate increased from 5 to 13 cases per 100,000 inhabitants, yet the prevalence was five times less than that of southern Africa in 2018 (Hamdi et al., 2021). In Eastern, Central, and Western Africa, it was observed in 2018 in Central Africa that the PCa incidence rate reached 35 cases per 100,000 inhabitants. Genetics is the first factor that may explain the increased risk of cancer occurrence in Sub-Saharan Africa, without ignoring the potential carcinogenic impact of environmental and lifestyle factors. In Southern Africa, PCa is considered the deadliest cancer for men (Bray et al., 2018). In Central Africa, PCa is the most common cancer and the deadliest cancer in men. Additionally, it is the second leading cause of cancer-related death after lung cancer in men in Northern Africa (Hamdi et al., 2021). Most PCa cases were recorded in southern Africa, where the number of new cases increased dramatically from 2002 (40,5 per 100,000 habitants) to 2018 (64,1 per 100,000) (Hamdi et al., 2021). The International Agency for Research on *Cancer* (IARC) expected 57,048 deaths from PCa in 2030, with approximately 28,006 deaths in 2010 in Africa, which tends to be a real health problem on this continent (Wang et al., 2018). This would represent, in the next 2 decades, a 104% increase in the number of PCa deaths in Africa. This may be due to a lack of screening, underdiagnosis, or treatment. The International Consortium of PCa Genetics (ICPCG) was founded to compensate for and provide systematic research in this field (Kral et al., 2011).

To quantify PCa burden in Africa, extensive research and effective cancer registration are crucial. Adeloye et al. (2016) provided, based on available data, a continent-wide incidence rate of PCa by reviewing the literature (9,766 records, with 40 studies) on PCa spreading across 16 African countries. The research was conducted from January 1980 to June 2015. The authors identified an incidence of 22.0 per 100,000 population and reported a median incidence rate of 19.5 per 100,000 population. The authors also observed an increase in PCa incidence with advancing age (Adeloye et al., 2016).

Likewise, Seraphin et al. (2021) analyzed registry data from 12 population-based cancer registries across 11 sub-Saharan African (SSA) countries. The data analyzed were approximately 13,170 incident PCa cases in men aged 40 years or above, with at least a 10-year time span of comparable data. The authors pointed out an increase in age-standardized incidence rates (ASR) and cumulative risks (CR) over time in all registries. A steady increase in incidence with age and during successive periods was observed. These findings underscored that in SSA populations, PCa incidence rates are rising, often very rapidly (Seraphin et al., 2021).

The best-known risk factors that have been characterized include age, diet (World Cancer Research Fund/American Institute for Cancer Research (WCRF/AICR), 2018), smoking (Plaskon et al., 2003), and genetic predispositions (Benafif and Eeles, 2016). Gene mutations are common in healthy cells but do not necessarily lead to malignant cells. However, sometimes, the reparation processes cannot correct the errors at the DNA level, and the defense system cannot eliminate the existing malignant cells. This can result in unregulated proliferation, leading to oncological disease status (Kral et al., 2011).

PCa can be segmented for practical purposes into three categories: 85% sporadic, 15% hereditary/familial (Kral et al., 2011). Some families’ high prevalence and early onset of this cancer led to extensive genome studies revealing PCa susceptibility genes or loci (Kral et al., 2011). The most common pathogenic germline variants involved in PCa include BReast CAncer gene 1 (BRCA1), BReast CAncer gene 2 (BRCA2), Homeobox Protein Hox-B13 (HoxB13), and Checkpoint Kinase 2 (CHEK2) (Wang et al., 2018). The risk of this disease increased in men of African Americans, regardless of their present environment, which further supports a robust ethnic association.

PCa often has a slow course that requires active monitoring and may not show any symptoms in the early stage (Rawla, 2019). PCa is becoming increasingly a problem of great concern to the public in Africa because most newly diagnosed cases are in the late stage, with poor prognosis (Giona et al., 2021). African Americans are nearly 2.5 times more likely to die from PCa than other populations and are more likely to be diagnosed with this disease in the United States (Rebbeck et al., 2013). This review raises the issue of the limited prostate cancer genomic and -omics studies conducted in Afro-native populations and highlights the challenges that the African people face, limiting them from conducting relevant studies. We also define the common variants associated with PCa in Afro-native populations and how they differ from European populations.

## PCa omics studies in African descent

PCa has been the subject of a number of omics studies, including genomics, proteomics, transcriptomics, and epigenomics. Genomic studies have been the most applied compared to other omics strategies. We will first briefly describe these PCa-omics studies before devoting a full section to PCa genomics.

The recent development of systemic analytical-omics strategies, namely, genomics, transcriptomics, proteomics, metabolomics, epigenomics, and metagenomics, has contributed to early and accurate cancer detection and novel therapeutic strategies by revealing key mutations and molecular pathways of tumor growth and progression (Yoo et al., 2018). However, omics studies in Africa are limited. From the entire African continent, only a few PCa studies in genomics, metagenomics, and proteomics have been carried out in South Africa, Ghana, Tunisia, and Uganda (Table 1). When searching for research papers on omics in Africa, only those that included African populations or separate analyses on an African population were included in this review.

**TABLE 1:** List of African studies on prostate cancer omics.

Omics method	Year of publication	Country	References
Genomics	2015	South Africa	McCrow et al. (2016)
Genomics	2018	South Africa	Jaratlerdsiri et al. (2018)
GWAS	2015	South Africa	Fernandez et al. (2015)
GWAS	2014	Ghana	Cook et al. (2014b)
GWAS	2013	Tunisia	Shan et al. (2013b)
Metagenomics	2019	South Africa	Feng et al. (2019)
Proteomics	2015	South Africa	Adeola et al. (2015)

Extensive research is needed to understand the unknown contribution of omics variation to African PCa racial disparity, unlike non-African studies. Few African studies on PCa omics have been conducted, mainly on genomics, proteomics, and metagenomics (Adeola et al., 2015; McCrow et al., 2016; Jaratlerdsiri et al., 2018; Feng et al., 2019). Jaratlerdsiri et al. performed the first tumor-normal paired whole-genome sequencing for high-risk PCa in South African men, which revealed an elevated tumor mutational burden that can be used to improve patient outcomes in Africa (Jaratlerdsiri et al., 2018). Adeola et al. conducted a proteomic study on multiethnic cohorts of South African patients, revealing seventy-three new urinary protein biomarkers for PCa *via* label-free mass spectrometry. This study demonstrated that urinary proteomics, which is less complex to analyze than other proteomes derived from serum, tissue or cells, is a concrete track to discover new prostate cancer biomarkers (Adeola et al., 2015). In another study, Feng et al. performed a metagenomic analysis of prostate tumors from 22 men and showed their microbial content. Bacterial richness within the African vs. non‐African samples was observed throughout this study with an abundance of anaerobic bacteria. Additionally, pathogenic microbes may play a role in oncogenic transformation within the prostate microenvironment (Feng et al., 2019).

Epigenetics is known as the study of heritable changes in gene expression mediated by factors other than alterations in DNA sequences. DNA methylation and histone modification, two prominent epigenetic processes, have been found to play significant roles in prostate cancer growth and metastasis (Albany et al., 2011). In African American (AA) men, the correlation between differential DNA methylation and PCa recurrence and clinical features of aggressive disease has been identified by Rubicz et al. (2019). Tumor tissues from 76 AA PCa patients with radical prostatectomy were profiled for epigenome-wide DNA methylation levels. Patients with PCa recurrence have been identified, and twenty-three CpG methylated sites have been found in both patients with and without recurrence, including CpGs in glucokinase (GCK), cyclin-dependent kinase like 2 (CDKL2), PR/SET domain 13 (PRDM13), and zinc finger RNA binding protein 2 (ZFR2). In the three groups of patients, higher vs. lower tumor aggressiveness, metastatic PCa vs. no recurrence and regional vs. local tumor stage, the CpGs were also found to be differentially methylated (Rubicz et al., 2019). Likewise, the objective of the work of Moses-Fynn et al. (2018) was to examine the clinical significance of promoter gene DNA methylation modifications in whole blood from AA men with PCa (Moses-Fynn et al., 2018). High-throughput pyrosequencing analysis was used to quantify, in blood DNA obtained from PCa and healthy control cases, the percentage of DNA methylation levels in a panel of eight genes (retinoic acid receptor beta2 (RARβ2), TIMP metallopeptidase inhibitor 3 (TIMP3), secreted protein acidic and cysteine rich (SPARC), cadherin 13 (CDH13), HIN1, long interspersed element class 1 (LINE1), cytochrome B5 reductase 2 (CYB5R2) and dopamine receptor D2 (DRD2)). Notable differences in DNA methylation levels were marked between the PCa compared to control cases in six of the tested genes. Considerable correlation was identified between DNA methylation of TIMP3 loci and age of all analyzed cases. The authors observed an inverse correlation between CDH13 methylation and serum vitamin D levels, while TIMP3 methylation and DRD2 methylation demonstrated an inverse correlation with supplementary vitamin D in PCa cancer cases. They also revealed a direct correlation of SPARC and RARβ2 locus methylation with PSA levels only in the controls. These data suggest that for AA PCa patients, promoter methylation occurs more abundantly in their blood and is associated with several clinicopathological characteristics in AA men with PCa (Moses-Fynn et al., 2018).

Proteomic profiling is an important component of functional genomics and plays a key role in determining different alterations associated with disease processes in addition to explaining the functional pathways in normal cells (Ahram et al., 2002). The first and most commonly used test for the early detection and monitoring of PCa worldwide is the prostate-specific antigen. To identify tumor-associated antigens (TAAs) of PCa, Dai et al. (2016) used a serological proteome analysis (SERPA) approach (Dai et al., 2016). The authors identified and characterized a TAA in PCa called nucleophosmin 1 (NPM1). The higher level of anti-NPM1 antibody in PCa compared to the one detected in benign prostatic hyperplasia (BPH) patients and healthy individuals. A total of 97.1% of PCa patients at an early stage were identified correctly using serum measurements of anti-NPM1 antibody and PSA. Furthermore, it has been found that after surgical treatment of early-stage PCa, the anti-NPM1 antibody levels in these patients at an early stage have been increased. These outcomes indicated that NPM1 may play a potential role as a prognostic biomarker for PCa (Dai et al., 2016).

Few studies have been conducted to investigate the role of cancer testis antigens (CTAs) in PCa in men of African descent. Therefore, Adeola et al. (2016) examined the role of 123 tumor-associated antigens (TAAs) in blood samples from a South African PCa, benign prostatic hyperplasia (BPH) and disease control (DC) cohort using an antigen microarray platform (Adeola et al., 2016). A total of 41 potential diagnostic/therapeutic antigen biomarkers for PCa have been identified. MAGE family member B1 (MAGEB1) and PRKCZ were highly expressed, and four antigens, G antigen 1 (GAGE1), rhinophilin-associated tail protein 1 (ROPN1), sperm protein associated with the nucleus, X-linked, family member A1 (SPANXA1) and protein kinase C zeta (PRKCZ), had higher autoantibody titres in PCa serum than in BPH serum. Additionally, in the disease control group, the p53 S46A and p53 S15A genes were found to have higher expression. Hence, it has been proven that fibroblast growth factor receptor 2 (FGFR2), collagen type VI alpha one chain (COL6A1) and calmodulin 1 (CALM1) are biomarkers of PCa. Similar enrichment was revealed by functional pathway annotation of identified biomarkers at the proteomic and genomic levels, aside with ethnic variations. Henceforth, cancer antigen arrays have been revealed to be an important discovery of diagnostic and immunotherapeutic antigen biomarkers (Adeola et al., 2016).

Similarly, Adeola et al. (2015) used direct, mass spectrometry-based proteomics to identify twenty potential urinary biomarkers of PCa in South African patients among 1,102 and 5,595 protein groups and nonredundant peptides (Adeola et al., 2015). ±2 SD fold differences were found in 17 proteins, and twenty promising biomarkers were identified. A total of 1,545 and 9,991 nonredundant protein clusters and peptides were obtained after an extensive analysis of 45 individual samples. Potential PCa biomarkers were found among seventy-three (McDonald et al., 2014) protein clusters that included putative PCa biomarkers and demonstrated ethnic trends within the PCa cohort. The outcomes highlight that urinary proteomics is an encouraging method that may serve as a potential way to discover more PCa biomarkers and a promising source of ethnic-related biomarkers of PCa (Adeola et al., 2015).

Panigrahi et al. (2019) studied the discovery of a serum exosome-based protein signature that could be utilized to detect PCa in AA males earlier and provide a better prognosis (Panigrahi et al., 2019). The exosome concentration in the sera of AA men with PCa was 3.2 times higher than that in healthy individuals, according to nanoparticle tracking analyses. Seven unique and fifty-five overlapping proteins were revealed using mass spectrometry-based proteome analysis of serum exosomes in AA patients with PCa vs. healthy people. In addition, an inflammatory acute phase response signaling pathway was found to be a significant pathway linked to proteins in exosomes from AA PCa patients. Furthermore, proteomic investigations revealed that the loading in exosomes from AA with PCa was increased by protein isoform two compared to that in exosomes from race-matched healthy persons, but it led to lower loading in exosomes from Caucasian males with PCa. Overall, these findings confirmed the use of serum exosomes to assess the inflammatory phenotype and identify unique and new biomarkers linked to PCa in AA men without intrusive testing (Panigrahi et al., 2019).

Because there is no signature of immune reactivity for PCa, Weiner et al. (2021) examined immune profiles based on a large study that has been conducted in more than 1,300 patient samples annotated by race or genetic ancestry. Markers of natural killer (NK) cell activity, plasma cell infiltration and IgG expression have been found to be increased by prostate tumors in African men. These outcomes are promising and suggest that plasma cells may be potential biomarkers of immunotherapy response and drivers of PCa immunoreceptivity. (Weiner et al., 2021).

Hardiman et al. (2016) explored the vitamin D3 supplementation effects and the prostate transcriptome in 10 AA and 17 EA (Hardiman et al., 2016). Fourteen participants were given vitamin D3, and 13 were given a placebo for 2 months prior to surgery. The findings revealed that AA has greater levels of gene expression associated with immune response and inflammation. According to systems-level analysis, the disease evolution in AA may affect inflammatory processes (Hardiman et al., 2016). Furthermore, Hardiman et al. (2021) explored cellular stress responses and molecular profiles using a systematic approach in a key group of AA and EA males: men who had their prostates biopsied. They used high-throughput RNA sequencing (RNA-seq) to examine the prostate transcriptome from a single biopsy core. Different pathways were found to be altered, including the phosphatidylinositol three kinase (PI3K)/protein kinase B (PI3K-Akt) signaling pathway. The difference in the various genes expressed between EA and AA provided a unique genomic signature for each group (Hardiman et al., 2021).

## Prostate cancer genomics worldwide

Comprehensive multiomics profiles of cancer patients have been generated globally as a result of several efforts and initiatives (Huang et al., 2017). Fifty-eight percent of PCa cases have a heritable origin (Wu and Gu, 2016). The risk of men with a family history of PCa in first-degree relatives is 2.1-fold higher than that of men without a family history (Frank et al., 2015). Extensive genomics studies have been conducted worldwide in North America and Europe (Dean and Lou, 2013).

The spectrum of genomic lesions and changes in this disease are very diverse. This heterogeneity complicates PCa risk stratification and the selection of suitable and efficient management strategies. This spectrum includes single nucleotide polymorphisms (SNPs) (Allemailem et al., 2021), copy number variations (CNVs) (Barbieri et al., 2013), translocations (Barbieri et al., 2013), duplications (Quigley et al., 2018) and transversions (Lindquist et al., 2016), along with INDELs (small insertions or deletions) that can lead to frameshifts deleterious to the gene product (Barbieri et al., 2013). One of the most recurrent genome modifications is alterations affecting specific signaling pathways, such as tumorigenesis. ‘Phosphatase and TENsin homolog’ (PTEN) is the most common focal chromosomal loss in PCa genomes, and overall, PTEN harbors low mutation rates in PCa compared to other cancers. In addition, point mutations are most frequent in speckle-type POZ protein (SPOP) in localized cancers, including PCa (Taylor et al., 2010; Barbieri et al., 2012; Lindberg et al., 2013).

Extensive research is still needed to identify the normal range of variation for alleles at the GGC and CAG trinucleotide repeats of the androgen receptor (AR) gene, even though several studies have implicated these two loci in aggressive PCa. More particularly, there are few data on CAG and GGC allelic variation and linkage disequilibrium (LD), including data in six diverse populations from Africa, Asia, and North America that were analyzed by Kittles et al. (2001). It has been shown, as a result of this study, that populations of African descent possess significantly shorter CAG and GGC alleles than non-African populations. Additionally, African Americans (AA), including Africans from Nigeria and Sierra Leone, possess significant allelic diversity for both markers compared to other populations. Significant LD was revealed in healthy AA. The latter may be due to recurrent gene flow from diverse West African populations, migration of AA, and admixture with European Americans. Typing SNPs within the gene and performing haplotype analyses would play key roles in the identification of these haplotypes that were found to be significantly associated with high risk for PCa (Kittles et al., 2001).

Another critical example concerns amplifications and mutations of AR gene that have been found to appear following therapies targeting the androgen axis and metastatic lesions (Baca et al., 2013). Moreover, gene fusions involving the transcription factor family ETS (most commonly transmembrane protease serine 2:v-ets erythroblastosis virus E26 oncogene homolog (TMPRSS2-ERG)) have been identified in 50% of prostate-specific antigen (PSA)-screened PCa cases. Whole-genome sequencing studies (WGS) have revealed that a large number of chromosomal rearrangements, including complex patterns of rearrangement that have been termed “chromoplexy”, involve some known cancer-associated genes (Baca et al., 2013; Weischenfeldt et al., 2013).

In addition, other omics studies have been conducted in developed countries and have revealed significant variants and genomic alterations associated with genetic ancestry (Petrovics et al., 2015; Yuan et al., 2020). Examples of PCa transcriptomic analyses showed that these tumors could be subclassified based on gene expression and somatic copy number alteration (SCNA) signatures. These are promising subclasses identified in PCa associated with a specific genomic alteration pattern, which may lead to the successful prediction of some aggressive features of PCa (Lapointe et al., 2004; Lapointe et al., 2007; Taylor et al., 2010; Barbieri and Tomlins, 2014). However, we should keep in mind that, unlike other cancers, small somatic coding mutations are relatively rare in this type of cancer. At the same time, recurrent noncoding aberrations and more extensive genomic rearrangements are more common (Hayes et al., 2018).

Several genome-wide association studies (GWAS) has been reviewed in Europe and North America (Benafif et al., 2018). For example, Schumacher et al. generated a polygenic risk score (PRS) of 140,000 cases and controls of European ancestry to classify individuals into high- and low-risk categories (Schumacher et al., 2018). Shang et al., 2013 reported a mutation that was one of the significant findings in 24 213 Cases of PCa and 73 631 Controls from populations of European descent (mainly Canada and Northern Europe), which is the missense G84E variant in HOXB13 (Shang et al., 2013). Gudmundsson et al., 2009 reported four variants associated with susceptibility to PCa from a PCa genome-wide association follow-on study. The variants are: rs10934853 [A] on 3q21.3; rs16902094 [G] and rs445114 [T] on 8q24.21; and rs8102476 [C] on 19q13.2 (Gudmundsson et al., 2009a). The genomic region 8q24 has been detected as a critical region that has been found to be instantly implicated in PCa and other cancers over 200 independent PCa risk-associated loci through different studies (Freedman et al., 2006; Murphy et al., 2012; Fernandez et al., 2015). Part of the high incidence detected in African populations may be due to the risk variants at 8q24, which are more common in men of African descent and could explain the underlying genetic contribution to racial and ethnic disparities of PCa (Haiman et al., 2011a).

Ewing et al. (2012) sequenced germline DNA from 94 unrelated patients with PCa in order to investigate more than 200 genes in the 17q21–22 region. To determine the frequency of the discovered mutations, they tested relatives, additional case individuals, and control subjects. A rare but frequent mutation (G84E) in the homeobox transcription factor gene HOXB13 (rs138213197), which is crucial for prostate development, was found in four families of probands. In these four families, there were 18 men with PCa, and all of them had the mutation. In 5,083 unrelated people of European heritage with PCa, the carrier rate of the G84E mutation was elevated by a factor of almost 20, with the mutation being discovered in 72 subjects (1.4%) as opposed to one in 1,401 control participants. Compared to males with late-onset, nonfamilial PCa, men with early-onset, familial PCa had a significantly higher prevalence of the mutation. Even while the HOXB13 G84E mutation only makes up a small portion of all PCa, this discovery has ramifications for estimating the risk of PCa and could offer new mechanistic insights into this frequent cancer (Ewing et al., 2012). By the same token, to examine the association of the recurrent mutation (G84E) in HOXB13 with PCa risk in a large international sample of PCa families, Xu et al. (2013) genotyped this mutation and 14 other SNPs in or flanking HOXB13 in 2,443 PCa families recruited by the International Consortium for Prostate *Cancer* Genetics (ICPCG). 112 families (4.6%), all of European descent, with PCa had at least one mutation carrier. Men with a diagnosis of PCa were more likely to have the G84E mutation among carrier families than men without. G84E was found to be considerably over-transmitted from parents to affected children in a family-based association test. The G84E mutation was also found in the same haplotype in 95% of carriers, according to analysis of the markers surrounding it, which is compatible with a founder effect. These results confirm the relationship between the HOXB13 G84E mutation and PCa risk by showing that it is present in about 5% of PCa families, mostly of European descent (Xu et al., 2013). Likewise, Chen et al. (2013) aimed to compare the HOXB13 G84E mutation frequency among subjects of different races/ethnicities from various geographic regions in the world and to assess its risk for developing PCa, in the Reduction by Dutasteride of Prostate *Cancer* Events (REDUCE) trial. All of the 3,508 participants underwent initial negative prostate biopsies and were retested for PCa at years 2 and 4. Only Caucasians were found to carry the G84E mutation, with Northern Europe having the highest carrier frequency (1.06%), followed by Western Europe (0.60%), and North America (0.31%). In Southern Europe, Eastern Europe, Latin America, Australia, and South Africa, no mutation carrier was found. G84E mutation frequency in Caucasians was 0.99% in positive biopsy cases and 0.24% in negative biopsy subjects, respectively. Subjects with a positive family history were considerably more likely to have positive biopsy results than those without. The PCa detection rate was 53.8% among the 13 mutation carriers and 22.0% among the 3,186 non-carriers over the 4-year follow-up; the relative risk was 2.45. In conclusion, the relatively recent G84E mutation of HOXB13, which most likely happened in Northern Europe, greatly raises the risk for PCa (Chen et al., 2013).

18 SNPs in a 1-Mb region at 8q24 were examined by Zheng et al. (2007) to determine their relationships with the risk of PCa in 1,563 case patients and 576 control participants of European American ancestry. They discovered several SNPs in a 50 kb region (known as locus 1) that are in linkage disequilibrium with rs1447295 at 8q24, a previously reported risk-related SNP, but were more significantly associated with PCa risk in this study sample. They also identified a novel susceptibility SNP, rs6983267, at a second locus (locus 2) that is approximately 70 kb centromeric of rs1447295 and in linkage equilibrium with, and independent of, locus 1. Risk alleles at locus two were widespread in the group under study. A third locus at 8q24, roughly 400 kb centromeric to locus 2, was also found by the scientists, and it was statistically significantly linked to PCa risk. These findings demonstrated the existence of three distinct genetic risk factors for PCa at loci around 8q24 (Zheng et al., 2007). In the same context, Amundadottir et al. (2006) reported a variant on chromosome 8q24, a region initially identified through a study of Icelandic families. In three case-control series of European ancestry from Iceland, Sweden, and the US, allele eight of the microsatellite DG8S737 and rs1447295 A were linked to PCa. The DG8S737 allele’s estimated odds ratio (OR) is 1.62. A population attributable risk (PAR) of 8% is produced by the fact that 13% of the general population and about 19% of affected men carry at least one copy of the gene. A case-control study of African Americans with a similar OR, 41% of affected people, and 30% of the population as carriers, likewise confirmed the connection. These results may explain why African American men have a higher incidence of PCa than those with European heritage (Amundadottir et al., 2006).

Over 23,000 Icelanders were the subjects of a genome-wide SNP association analysis on PCa by Gudmundsson et al. (2008), which was followed by a replication study with over 15,500 people from Europe and the United States. The newly discovered variants rs5945572 on Xp11.22 and rs721048 on 2p15 have been linked to PCa. It was also revealed that more aggressive forms of the disease exhibit a substantially stronger link with the 2p15 mutation than do less aggressive types (Gudmundsson et al., 2008). On the other hand, using 1,501 Icelandic males with PCa and 11,290 controls, Gudmundsson et al. (2007) conducted a genome-wide association scan to look for sequence variants that increase the risk of PCa. Follow-up studies with three more case-control groups confirmed a relationship of two chromosome 17 polymorphisms with the illness. These two variations, which are 33 Mb apart, are located in a region that has previously been connected to PCa by family-based linkage studies. The two common variants on chromosome 17q, rs4430796 A and rs1859962 G, contribute to the risk of PCa. However, it has been shown that one of the variations (rs4430796 A) in TCF2 (HNF1beta) confers protection against type 2 diabetes (Gudmundsson et al., 2007).

Takata et al. (2010) performed a GWAS and a replication study using 4,584 Japanese men with PCa and 8,801 control subjects. From the thirty-one associated SNPs reported in previous GWAS in European populations, they confirmed the association of nine SNPs at *p* < 1.0 × 10–7 and ten SNPs at *p* < 0.05 in the Japanese population. The remaining 12 SNPs showed no association. In addition, they reported five new PCa sensitivity loci at 5p15, GPRC6A/RFX6, 13q22, C2orf43, and FOXP4. These findings advance our understanding of the genetic basis of prostate carcinogenesis and also highlight the genetic heterogeneity of PCa susceptibility among different ethnic populations (Takata et al., 2010).

In the Chinese population, Zheng et al. (2007) investigated the variations connected to PCa risk. In a community-based case-control research from Shanghai, which included 288 PCa cases and 155 population controls, they assessed 17 PCa susceptibility loci. Two of the 17 loci were significantly connected with PCa risk in this sample after age adjustment, while the remaining 15 loci were suggestively related. The regions of chromosome 8q24 with the highest relationships were Region 2 (rs1016343) and Region 1 (rs1009015); additional SNPs examined in these two 8q24 regions also showed significance. This work demonstrates that several PCa risk loci discovered in European countries by GWAS are also connected to PCa risk in Chinese men, a group with low cancer incidence and a high percentage of clinically significant malignancies (Zheng et al., 2007).

Ren et al. (2018) sequenced the entire genome and transcriptome of matched tumor-benign tissues from 65 Chinese PCa patients who had never received treatment. In a subsequent cohort of 145 PCa, targeted deep sequencing of 293 PCa-relevant genes was carried out. The researchers showed that in Chinese individuals, a high frequency of CHD1 deletion was associated with a low frequency of TMPRSS2-ERG fusion and a comparatively high proportion of mutations in the genes that code for the androgen receptor upstream activator. Additionally, they discovered five probable clustered deleted tumor suppressor genes and provided experimental and clinical proof that PCDH9, a novel tumor suppressor gene with prognostic value in PCa that is deleted or lost in about 23% of tumors, works as a tumor suppressor gene. Additionally, many genes involved in the axon guidance pathway were dysregulated, with 17% of tumors exhibiting gain or amplification of the PLXNA1 gene (Ren et al., 2018).

In recent years, computational multiomics approaches have been suggested as promising tools instead of singleomics approaches. Likewise, these multiomics tools and methods typically aim at classifying patients into cancer subtypes and gaining steps toward the pattern of precision medicine (Shen et al., 2009; Lock et al., 2013; List et al., 2014; Ray et al., 2014; Gligorijević et al., 2016).

## Prostate cancer genomics of African descent

### Somatic/tumor mutations

As we mentioned above, due to genomic factors, men of African Americans show higher PCa mortality and incidence than men of another ancestry (Yuan et al., 2020). However, there is a significant lack of knowledge about the factors contributing to disparities in PCa incidence between men of African and European descent (Zeigler-Johnson et al., 2008).

To investigate this hypothesis, Yuan et al. evaluated the genomic profiles of 61 African Americans and 414 European American (EA) cases. Considerable differences were highlighted in the frequency of SPOP gene mutations (20.3% African Americans vs. 10.0% EA), PTEN gene deletions/losses (11.5% African Americans vs. 30.2% EA), and TMPRSS2-ERG gene fusions (29.3% African Americans vs. 39.6% EA). These genomic analysis results indicated that the differences observed might be due to racial disparities in the incidence of PCa (Yuan et al., 2020). Furthermore, to study the genomic alterations associated with race, the frequencies of somatic alterations in a cohort of AA and EA PCa patients were compared by Koga et al. Focal deletions in ETS Variant Transcription Factor 3 (ETV3), His-tone-lysine N-methyltransferase 2D (KMT2D) truncations, mutations in Zinc finger homeobox 3 (ZFHX3), and Cyclin D1 (CCND1) amplifications in early-stage PCa were more abundant in AA patients. On the other hand, deletions in PTEN and rearrangements in the TMPRSS2-ERG gene were more frequent in AA cases. In contrast, genomic alterations in cyclin-dependent kinase 12 (CDK12) and in androgen receptor (AR) were found to be similar between AA and EA patients (Koga et al., 2020). Furthermore, Petrovic et al. performed a systematic whole-genome analysis to uncover the alterations that differentiate the PCa genome of AA and Caucasian Americans (CA). A recurrent deletion on chromosome 3q13.31 has been discovered on the Limbic System-Associated Membrane Protein (LSAMP) locus, which is frequent in AA men PCa patients. In addition, interchromosomal rearrangements were significantly more prevalent in AA tumors than in CA tumors. However, ETS transcription factor ERG (ERG) and PTEN PCa alterations were remarkably lower in AA patients than in CA patients. These outcomes revealed differentially distributed somatic mutations in PCa across ancestral groups (Petrovics et al., 2015). In another study conducted by Yamoah et al., the authors compared gene expression in a cohort of PCa patients, including Native African men (NAM), European American men (EAM), and African American men (AAM). As expected, TMPRSS2-ERG fusion was found in 56% of EAM tumors, while ERG was detected in 24% of AAM and 17% of NAM tumors. Serine peptidase inhibitor Kazal type 1 (SPINK1) expression was fourfold higher in NAM than in EAM. These findings uncover significant transcriptomic differences between NAM and their European counterparts (Yamoah et al., 2020).

In European men, TMPRSS2-ERG gene fusion is considered the most common genomic alteration detected in carcinogenesis among this population. However, this gene fusion is less frequently detected in AA men, even though they mostly present with more advanced disease. In the study of Blackburn et al. (2019), reverse transcription polymerase chain reaction (RTPCR) was used on the RNA of 181 prostate Black South African men (94 PCa vs. 87 controls) to screen the TMPRSS2‐ERG fusion in this cohort. A frequency of 13% for TMPRSS2‐ERG has been identified, which may explain the link between TMPRSS2‐ERG fusion status and PCa racial health disparity (Akinloye et al., 2011).

### Germline genetic variation

Within the last few years, genomics studies have taken the lead over genetic studies because they have become feasible from cost and effectiveness standpoints. Genomics is basically the large-scale study of a set of genes and involves several areas of research, including genomics (WGS, GWAS), transcriptomics and metagenomics. GWAS has recently become one of the most used approaches that can detect associations between (germline) genetic variants and traits in PCa samples and other cancers from various populations (McDonald et al., 2014; Visscher et al., 2017).

Ntais et al. (2003) carried out a meta-analysis of several studies with CYP17 genotyping on a large cohort of patients (2,402) and controls (1755). These analyses indicated that the T-to-C polymorphism in the 5′-promoter region of the CYP17 gene cannot be considered a high-risk factor for sporadic PCa in different populations, especially in individuals of European descent (Ntais et al., 2003).

Chang et al. used four GWAS datasets (2 of African descendants and two of non-Hispanic whites) to explore the association of stemness-related gene variations and racial disparities in predisposition to PCa. They identified 32 SNPs in five genes significantly associated with PCa risk. Six SNPs in three genes and eight epidermal growth factor receptor (EGFR) SNPs showed diversity in susceptibility between African descendants and non-Hispanic white groups. In addition, only in African descendants were 13 SNPs in MET and one in aldehyde dehydrogenase one family member A1 (ALDH1A1). These SNPs were predicted to regulate RNA splicing based on *in silico* analyses. These findings identify that these RNA splicing-regulatory SNPs are potential biomarkers in PCa risk, especially the molecular mechanisms level of racial disparities (Wang et al., 2017). Zeigler-Johnson et al. analyzed 47 GWAS-identified loci from 7,788 PCa patient cases and controls. They pointed out considerable associations for SNPs rs5945619 in the Nudix Hydrolase 10 (NUDT10) gene, rs10993994 in the microseminoprotein beta (MSMB) gene, rs7931342 at 11q13, and rs10486567 in the JAZF zinc finger 1 (JAZF1) gene. These associations were also identified in men of European descent (Chang et al., 2011).

Disparities in incidence and outcomes are hallmarks of the global pattern of PCa. This significantly affects African men in terms of mortality, morbidity, and incidence (Zeigler-Johnson et al., 2008). Currently, there are considerable gaps in our knowledge about the factors that predict disparities in PCa between men of African and European descent. This is true in the United States, the Caribbean, and even Africa (Zeigler-Johnson et al., 2008). Studies involving AA men in the primary or replicate samples have detected PCa susceptibility variants associated with African Americans (Tan et al., 2018). Because African and AA men have common ancestry, knowledge of PCa in African men and comparisons of PCa in AA men and men of the African Diaspora may provide valuable data about the causes, treatment, and prevention of the disease. International comparisons of PCa rates are complicated by differences in reporting systems, cancer diagnosis, and screening (Zeigler-Johnson et al., 2008). In a study carried out by Zeigler-Johnson et al., the authors highlighted essential differences in the frequency of the V89 L variant in the steroid five alpha-reductase 2 (SRD5A2) gene by ethnicity, with an L allele frequency of 18% in Senegalese, 19% in Ghanaians, and 27% in AA. Differences were also shown for the CYP3A4*1B allele, with *1B frequencies of 78% in Senegalese, 81% in Ghanaians, and 59% in AA. Africans and AA were inferred to be more likely than the other populations to have genotypes associated with a high risk of PCa at the SRD5A2 and CYP3A4 genes. This fact suggests a shared inherited predisposition to PCa (Zeigler-Johnson et al., 2002). A particular GWAS that focused on a sample of Ghanaian men provided a novel specific locus for low- and high-risk PCa at 10p14 (Cook et al., 2014a). Furthermore, nominal associations for rs75823044 at the 13q34 locus and for rs78554043 at 22q12.1 were identified in a cohort of Ugandan men (Du et al., 2018). Likewise, Fernandez et al. validated PCa susceptibility variants rs10993994 (10q11), rs6983267 (8q24), and rs7008482 (8q24) in 331 South African cases (Fernandez et al., 2015). The risk variants that are specific to men of African ancestry (MAA), including but not limited to South African, Ghanaian, Tobago men Afro-Caribbean, Ugandan and MAA, seem to be maintained by the 8q24 PCa susceptibility region (Lewis and Cropp, 2020). It has been found that the 8q24 locus was related to an increased risk for PCa and an earlier age of diagnosis among AA men (Schumacher et al., 2007). Other variants, including at least nine SNPs near 8q24, are independently associated with a risk of PCa in AA men (Tan et al., 2018). Darst et al. (2020) explored the rs72725854 variant at 8q24 variant in 143 cases from high-risk families, and 8,595 controls of African Americans. They found that rs72725854, an African Americans-specific risk variant, is more common in men with a family history of PCa and in those diagnosed with PCa at younger ages (Darst et al., 2020a). Murphy et al. (2012) genotyped ten 8q24 SNPs in 308 PCa cases vs. 469 controls of men from West African descent populations, notably Cameroon, Nigeria, and the Caribbean Island of Jamaica. A total of 380 samples from Sierra Leone were genotyped and served for a comparison of allele frequency. A significant association with PCa risk in West Africans of the following SNPs has been found: rs6983561, rs16901979, and rs7008482. The authors highlighted the absence of the SNP bd11934905 in 1,886 chromosomes from three West African ethnic groups, which is a specific African SNP of the A-risk allele. This result indicates that this allele is geographically restricted to specific ethnic groups. These findings indicate the significant association of 8q24 SNPs with PCa risk in men from Cameroon and Nigeria (Murphy et al., 2012).

Cropp et al. (2014) evaluated the relationship between 8q24 risk alleles that were reported previously and PCa in African Barbadian (AB) men. Among 532 AB PCa patients and 513 AB controls, ten SNPs were genotyped and/or imputed in the 8q24 region. The rs2124036 SNP was found to be significant in AB men for the homozygous C/C genotype. This outcome may show a founder effect or a selected SNP that does not mark the ancestral haplotype carrying the 8q24 risk allele(s) in this cohort. The authors carried out a meta-analysis on the AB population and the African Caribbean populations (Jamaica and Tobago) for SNPs rs1447295 and rs16901979. The outcomes of this meta-analysis were highly significant for the rs16901979 A allele (Cropp et al., 2014).

To date, only a few loci showing significant genome-wide association have been discovered in MAA. Recently, two potential association signals of novel genome-wide have been identified on chromosome regions 13q34 (rs75823044) and 22q12 (rs78554043) (Lewis and Cropp, 2020). Significant associations in MAA have also been revealed for SNP rs10993994 at the MSMB gene, rs10486567 at JAZF1 gene, rs5945619 at NUDT10 gene, and rs12418451 and rs7931342 at 11q13 chromosome location (Lewis and Cropp, 2020). In another study by Hooker et al., the authors evaluated 20 SNPs associated with PCa risk in a cohort of AA. They replicated five SNPs in the studied population: rs2735839 on 19q33.33 region, rs10896449 on 11q13.2, rs5945572 on Xp11.22, rs443076 on chromosome location 17q12, aside with bd 1934905 which is specific variant to West African Americans in region two of 8q24 (Hooker et al., 2010).

Chromosomal changes on 12q and 12p that exist during the progression of PCa have been reported previously. Bonilla et al. (2011) examined chromosome 12 to explore genetic risk factors that underlie PCa health disparities using ancestry informative markers (AIMs). They were able to differentiate between genomic regions of West African or European origin. The strongest signal of association was identified at the specific SNP rs12827748 on an abundantly expressed gene in PCa called the tumor suppressor (PAWR) gene. (Bonilla et al., 2011).

The observed differences in PCa rates within the United States could be explained by the genetic susceptibility loci that differ in allele abundance by ancestral population, especially that a large proportion of AA men are originally from West Africa (Du et al., 2018). In the research of Cook et al., the SNP (rs2993385 at 10p14) was the only replicated SNP at *p* < 0.05 between AA and West Africa. There are many primary causes that could explain the absence of ancestrally related genetic signals in the AA population (Du et al., 2018). There is a difference between the genomic map of AA men and African men because the AA population has received European gene flow for a long time with mixed ancestral percentages of 20% European, 5% Native American, and 75% West African (Bryc et al., 2010). In addition, there are significant differences in patterns of homozygosity, allele frequencies, private SNPs, and gene–environment interactions between African and AA populations (Du et al., 2018).

Over 33 loci have been identified, and the majority of these loci are in intergenic regions, while a few have known biological relevance to PCa (Yeager et al., 2007; Eeles et al., 2008; Thomas et al., 2008; Gudmundsson et al., 2009b; Eeles et al., 2010; Schumacher et al., 2011; Yeager et al., 2012) [66–72]. Multiple SNPs have been placed in different genomic regions. The strongest associations are to an area on chromosome location 8q24 nearest the MYC proto-oncogene and on chromosome 10 containing the rs10993994 SNP. One of the strongest SNP associations is in front of the MSMB gene (Eeles et al., 2008; Thomas et al., 2008; Guy et al., 2009). Currently, some antibodies against the MSMB protein, which is highly expressed in PCa, are used as biomarkers of PCa development (Ahn et al., 2012).

Some GWASs have shown that androgen plays a vital role in controlling the progression of normal prostate and in its ability to become an adenocarcinoma, which is termed the “androgen hypothesis” (Hsing et al., 2008; Yassin et al., 2019). Hence, many genetic variants that have been identified among African populations are variants that play a role in elevating circulating androgens, including those responsible for reducing clearance. Some of these polymorphisms have been identified in Morocco, Tunisia, Nigeria, South Africa, and Senegal, such as cytochrome P450 family three subfamily A member 4 (CYP3A4), cytochrome P450 family three subfamily A member 5 (CYP3A5) and cytochrome P450 family one subfamily A polypeptide 1 (CYP1A1) (Fernandez et al., 2010; Souiden et al., 2011; Fernandez et al., 2012; Souiden et al., 2012; Novillo et al., 2015). PCa genetic studies in North Africa and some sub-Saharan African populations reported in the AR gene, some alterations in CAG and GGN repeats as risk factors for this disease in these populations (Esteban et al., 2006; Akinloye et al., 2011). Moreover, glutathione S-transferase Mu 1 (GSTM1), UDP-glucuronosyltransferase, and sulfotransferase have been reported as genetic variation sites that increase African populations’ susceptibility to PCa in Tunisia and Algeria (Souiden et al., 2010; Fernández-Santander et al., 2016; Novillo et al., 2018).

Other PCa GWAS have investigated the PCa genome-wide genetic variations in three African countries, including Tunisia, Uganda, and Ghana (Shan et al., 2013a; Cook et al., 2014a; Du et al., 2018). The Ghanaian study discovered a total of 30 significant SNPs (Cook et al., 2014b). The Tunisian study revealed three regions on chromosomes 9, 17, and 22 containing 14 significant SNPs (Shan et al., 2013b). Furthermore, risk alleles on chromosomes 1, 6, 11, 13, 14, and 17 have been identified in the Ugandan study, and band 6p21.32 has been explored in the Ghanaian study (Du et al., 2018). These SNPs are highlighted in Supplementary Table S1.

Extensive studies that analyze the genomics of PCa in African populations are needed (Hindorff et al., 2018; Sirugo et al., 2019). Multiple GWAS have identified genetic correlations with PCa (Eeles et al., 2008; Haiman et al., 2011a; Schumacher et al., 2018). One of the few GWASs conducted was in Ghana, where unique PCa-associated SNPs of this population have been identified (Cook et al., 2014b). Haiman et al. performed a GWAS with 1,047,986 SNP markers to search for common risk alleles for PCa in 3,425 AA PCa patients and 3,290 AA male controls. The authors identified 17 novel associations in stage 1 and a risk variant on chromosome location 17q21. The risk allele that is rare in other populations was found to be in men of African Americans with a frequency of ∼5%. Despite this exciting finding, extensive research is needed to examine the role of this allele in PCa risk (Haiman et al., 2011a)**.** Fernandez et al. (2008) conducted research on 134 controls and 151 cases of South African-Colored men to explore the role of cyclooxygenase-2 enzyme (COX-2) variants in PCa in this population. The genotype frequencies of four SNPs have been detected in some controls and two alleles (21285 G and 21265 T) were found associated with an elevated risk of PCa. These results suggest a genetic association between variants in COX-2 and PCa risk in South African men (Akinloye et al., 2011).

Likewise, in 2015, a study among a cohort of patients in Senegal showed that hereditary factors or ancestral factors (black or African) did not affect the increase in the risk of PCa (Acheampong et al., 2021). However, this result seems to be applicable to other African populations (Acheampong et al., 2021). Recently, Conti et al. (2021) conducted a multiancestry meta-analysis of PCa GWAS studies (107,247 cases and 127,006 controls) and identified 86 new genetic risk variants independently associated with PCa risk in men from mixed populations including men from African Americans. However, there is still lack of studies conducted on African populations. The outcome of GWAS presents a step forward in better understanding the evidence of the heterogeneity of African populations (Schlebusch et al., 2017; Acheampong et al., 2021) and proves the need for further studies of the genomics of PCa and their contributions to racial disparities of this disease.

Chang et al. (2011) studied 7,788 PCa cases and controls of African descent with genotype data for 47 GWAS-identified loci. They detected significant associations for SNP rs10486567 at JAZF1, rs10993994 at MSMB, and rs5945572 and rs5945619 at NUDT10/11, rs12418451 and rs7931342 at 11q13. These associations were in the same direction and of similar magnitude as those reported in men of European descent. Significance was attained at all reported PCa susceptibility regions at chromosome 8q24, including associations reaching genome-wide significance in region 2. These susceptibility loci were originally also identified in non-African descent populations (Chang et al., 2011).

A linkage study was carried out by Brown et al. (2004) employing 33 AA PCa families from two different research groups. Utilizing markers that map to five PCa susceptibility loci, including HPC1 at 1q24–25, PCAP at 1q42–43, CAPB at 1p36, HPC20 on chromosome 20, and HPCX at Xq27–28, a total of 126 people (including 89 men with PCa) were genotyped. In the 20 families with male-to-male transmission and the 24 families with PCa diagnosis before to age 65, there was a greater evidence of linkage. Additionally, some PCa linkage evidence was found at markers corresponding to PCAP, HPC20, and HPCX (Brown et al., 2004).

Chung et al. (2014) resequenced a region comprising three PCa susceptibility loci as well as a locus linked with chronic lymphocytic leukemia to characterize variation in people of African Americans. The samples included 47 African-American students from Johns Hopkins University and 78 people from Ghana. A total of 1,838 SNPs were found. 285 of them were novel and had not yet been documented in any public database. They improved the linkage disequilibrium and recombination hotspots in the area using genotypes derived from sequencing, and they identified a collection of tag SNPs to be used in upcoming fine-mapping research (Chung et al., 2014).

Few GWAS have reported some phenotype-specific loci and genetic susceptibility variants and their association with PCa progression, survival, mortality, and clinical applications (Hicks et al., 2017). The clinical significance of some SNPs is depicted in Table 2.

**TABLE 2:** Clinical significance of SNPs associated with PCa in Afro-native populations.

SNP ID	Clinical significance	References
rs2993385	Not reported	—
rs72725854	African Americans–specific risk variant	Darst et al. (2020b)
rs114798100	Not reported	—
rs1053005	Associated with aggressive PCa	Kwon et al. (2011)
rs8074524	Associated with aggressive PCa	Kwon et al. (2011)
rs3809758	Associated with aggressive PCa	Kwon et al. (2011)
rs7045455	Located in the SMARCA2 gene which is downregulated in PCa tissues and confers the advantage of proliferation to PCa cells	Shan et al. (2013b)
rs12686439	Not reported	—
rs10810919	Located in the SMARCA2 gene which is downregulated in PCa tissues and confers the advantage of proliferation to PCa cells	Chen et al. (2014)
rs10963533	Not reported	—
rs10963540	Not reported	—
rs12601982	Associated with the expression of STAT5B gene which is active in both locally confined and advanced prostate cancer and critical for the growth of PC cells	Shan et al. (2013b)
rs8078731	Associated with the expression of STAT5B gene which is active in both locally confined and advanced prostate cancer and critical for the growth of PC cells	Shan et al. (2013b)
rs5750627	Associated with APOBEC3H that inhibits miRNA activity to promote cancer metastasis	Shan et al. (2013b)
rs6001173	Associated with APOBEC3H that inhibits miRNA activity to promote cancer metastasis	Shan et al. (2013b)
rs138702	Associated with SUN2 shown to be unrelated to cancer onset and progression	Shan et al. (2013b)
rs138712	Associated with SUN2 shown to be unrelated to cancer onset and progression	Shan et al. (2013b)
rs7008482	T/G	
rs6983267	Strongly associated with the risk of developing PCa	Hicks et al. (2017)
rs10993994	Strongly associated with both primary organ–confined and metastatic disease	Hicks et al. (2017)

## PCa-associated variants identified in African populations

Ethnicity has been associated with PCa risk (Petersen et al., 2019). The highest genomic risk for PCa has been found in West and South African Americans compared with other populations worldwide (Lachance et al., 2018; Petersen et al., 2019). Several PCa–associated variants have been discovered in some African countries, mainly in West Africa (Zeigler-Johnson et al., 2008), South Africa (Wang et al., 2018), and North Africa (Benafif and Eeles, 2016) (Supplementary Table S1). None of these SNPs overlapped in the different regions, indicating unique regional associated SNPs with PCa.

The alternate allele frequencies between Afro-native and Afro-American populations do not show differences. This may be attributed to the fact that the allele frequencies for the Afro-native people were calculated by including frequencies for African Americans and any individuals of African Americans (Phan et al., 2020). Thus, the Afro-native frequency is not representative of resident African populations, as resident African populations are rich in diversity (Choudhury et al., 2020). However, the frequencies between the European people and AA and Afro-native populations show differences. This demonstrates the genetic variation between these populations and highlights the need for more genetic studies in resident African populations (Table 3).

**TABLE 3:** Comparison of allele frequencies in various populations of the most significant (*p* < 0.05) SNPs associated with prostate cancer in Afro-native populations.

SNP ID	Allele/alt allele	Country	Alt allele freq afro-native	Alt allele freq African-american	Alt allele freq European
rs2993385	T |C	Ghana	0.2070	0.2102	0.52047
rs72725854	T	Uganda	0.0000	0.0000	0.00000
rs114798100	G	Uganda	0.0421	0.0406	0.00008
rs1053005	T/C	Tunisia	0.38680	0.38493	0.196873
rs8074524	C/T	Tunisia	0.3684	0.3673	0.18986
rs3809758	C/T	Tunisia	0.3816	0.3804	0.18341
rs7045455	T/C	Tunisia	0.8023	0.8044	0.94280
rs12686439	G/A	Tunisia	0.16013	0.1598	0.158199
rs10810919	T/C	Tunisia	0.9433	0.9425	0.97225
rs10963533	T/C	Tunisia	0.2931	0.2895	0.05832
rs10963540	G/A	Tunisia	0.7490	0.7513	0.936306
rs12601982	A/G	Tunisia	0.25851	0.25861	0.175087
rs8078731	A/T	Tunisia	0.2708	0.2701	0.16919
rs5750627	C/T	Tunisia	0.7111	0.7078	0.76307
rs6001173	C/T	Tunisia	0.5607	0.5607	0.68729
rs138702	T/A	Tunisia	0.9926	0.9923	0.83245
rs138712	A/G	Tunisia	0.5541	0.5538	0.70442
rs7008482	T/G	South Africa	0.8472	0.8436	0.313494
rs6983267	G/T	South Africa	0.11218	0.11451	0.487453
rs10993994	A/G	South Africa	0.3778	0.3782	0.596917

## Ethnic differences in genomics in Africa and PCa

Africa is an important region that is characterized by its complex population history and dramatic variation in diet, climate, and exposure to infectious disease, which has led to elevated levels of genetic and phenotypic variation in African populations (Campbell and Tishkoff, 2008). Interestingly, in research conducted by Yu et al., the results imply that Africans differ on average more among themselves than Eurasians (Yu et al., 2002).

PCa incidence and mortality rates are significantly related to race and ethnicity (Tan et al., 2018). GWASs of PCa have been remarkably successful in uncovering novel biological pathways and common genetic variants associated with its etiology (Haiman et al., 2011b).

Panz et al. (2001) carried out a study on 20 white and black PCa cases vs. 20 white and black healthy subjects to identify the number of polymorphic CAG repeat sequences in exon one of the AR gene of repeats and analyzed the polymorphic CAG repeat sequence in exon one of the AR gene as an indicator of PCa risk. Minor numbers of CAG repeats were observed in both patients and controls. Furthermore, a low number of repeats was observed in black and white patients with PCa aggressive disease compared to those with less aggressive PCa. The authors explained that these slight differences in the number of CAG repeats could not be considered as a potential indicator of PCa risk or aggressive disease but may contribute aside with other genetic and environmental risk factors into the risk and progression of PCa (Panz et al., 2001).

The PCa susceptibility loci differ by geography, race, and ethnicity, so it is more challenging to detect genetic associations. The African genomic variability is still unclear, and it is undeniable that several alleles that are rare or specific to men of African Americans still need to be explored (Rebbeck, 2017). In Africa, a few studies have examined variation in mortality or incidence from different cancers in “black” and “white” populations. However, very few studies have measured differences in risk between several ethnic groups living in the same country (Korir et al., 2017). In an attempt to characterize PCa locally, Dewar et al. studied the pathological and clinical features of 1,016 patients (162 were black, and 854 were colored, white, or Asian) from different racial backgrounds in South Africa undergoing prostate biopsy. In addition to the fact that they showed higher prostate-specific antigen (PSA) values, black patients more often presented with locally advanced cancer and had high-grade disease. Likewise, a case–control study in Kenya revealed substantial differences in cancer risk by ethnicity and race in residents of the Nairobi region (Korir et al., 2017).

The lack of replication across geography, ethnicity, and race may be due to various determinants, in particular, limited consideration of population-specific linkage disequilibrium, little capture of ethnic-specific alleles, and inadequate knowledge of population substructure. It is important to mention that genes do not only influence the underlying etiology of PCa. The various degrees of impact found across ethnicity and race aside with geography can also be due to contextual factors influencing susceptibility to PCa through genetic-environment interactions that may change by population (Rebbeck, 2017).

## Challenges and prospects in PCa genomics research in Africa

Genomic studies have been conducted on PCa in many countries and ethnic groups worldwide, while only a few have been conducted on African populations. The lack of extensive genomic studies in Africa is basically due to the limited research infrastructure and resources required to address cancer research needs and investigate the genomic heterogeneity of these populations. Moreover, many determinants, such as a lack of national cancer registries, limited access to health services, issues associated with social and cultural practices, and logistical and economic obstacles related to monitoring and conducting PCa research studies, play a role in the deteriorating health status of PCa cases within the continent (Tindall et al., 2014).

The main objective of the ongoing efforts in PCa omics, particularly genomics, is to achieve personalized medicine for patients of different origins around the globe. Therefore, further functional and genetic epidemiological research is needed on all continents, particularly in Africa, to gain a more in-depth understanding of the different genome variations and variants involved in the pathophysiology of PCa and thus to move toward targeted treatment programs suited to Africans. To make this a reality, large-scale genomic consortia must be set up to investigate the omics of African populations (El Jaddaoui et al., 2020; Acheampong et al., 2021). Of note, the global profiling of the genetic architecture of PCa cannot be straightforward without the analysis of the African genome.

There are disparities in research on PCa and cancer in general in African populations. Few countries in the African continent have the genomics research capacity to carry out research into cancer genomics mainly in East and South Africa. Kenya and Sudan are the only east African countries that possess a relatively powerful genomics and bioinformatics capacities and infrastructures to carry out this type of research including the Biosciences Eastern and Central Africa Hub Genomics and Bioinformatics Platform in Kenya that is equipped with high sequencing facilities (Fadlelmola, 2016). In addition to various bioinformatic networks, trainings and consortiums that have been lunched such as the H3 African Bioinformatics Network (h3ABioNet) in Africa (Mulder et al., 2016), the African Genomic Center (TAGC) launched in Cape Town, South Africa in 2018 (Shaffer et al., 2019), the doctoral training in bioinformatics provided in Uganda and Botswana through the Collaborative African Genomics Network (CAfGEN), the Eastern Africa Network of Bioinformatics Training (EANBiT) (Shaffer et al., 2019) and the African Academy of Sciences (AAS), and the African Organization for Research and Training in *Cancer* (AORTIC) (Rebbeck, 2020).

In order to address the disparities between Africa and other continents in terms of genomic research, the American Association for *Cancer* Research (AACR), the American Society of Clinical Oncology (ASCO), the American *Cancer* Society (ACS), and the National *Cancer* Institute (NCI) issued a position statement with detailed recommendations. These recommendations included changes to the funding of biomedical research for developing and underdeveloped nations (Fernandez, 2022). According to a recent report from the AORTIC conference, Africa needs to build data centers supported by the private sector, promote public-private partnerships, including biobanking, while being cautious about the commercialization of biospecimen and private partners’ desire for profit (Ayettey Anie et al., 2021).

Despite the efforts supplied by African researchers and different African imitories that aim to implement and improve the cancer genomic capacity on the African continent by integrating cancer genomics methods (CGMs), including genetic marker testing, customized arrays and genetic risk prediction models, to improve the representation of African populations in genomics databases or to implement precision medicine in African populations, the implementation of these CGMs is still challenging. Hence, the integration of the population genomic data of SSA would help with the use of big data and artificial intelligence as tools for potential approaches that would be beneficial as therapeutic and diagnostic approaches in cancer. Therefore, both the SSA population and Africans in the diaspora will benefit from the inclusion of CGMs due to their shared genomic resemblances and ancestry. Establishing centers of excellence for interdisciplinary cancer genomics in Africa could provide an excellent opportunity for collaboration between biotechnology companies, research institutions, and health systems and manage the cross-border sharing of cancer genomics data, investment, and laws on trade *via* regional initiatives (Munung et al., 2021).

The aim of a study conducted by Adedokun et al. (2016) was to investigate the author affiliations of genomic epidemiology articles published between 2004 and 2013 to help build and improve the poor genomics research capacity in SSA. The findings revealed South Africa (31.1%) as the most commonly studied population, followed by Ghana (10.6%) and Kenya (7.5%). Among all the papers published, 6.1% were related to cancer and one-tenth were related to noncommunicable diseases. Only 46.9% of the first authors were affiliated with an African institution. Most of the published papers in the genomic field were from South Africa since it has a powerful genomic infrastructure due to the great funding that this country receives (Adedokun et al., 2016).

Given that authorship is a proxy indicator of research capacity, Rotimi et al. (2021) analyzed and discussed the forms of authorship in Africa. Papers published between 1 January 1990, and 31 December 2019, and reporting cancer genomics in any African population were extracted and analyzed for these authorship forms. Only 0.016% of the worldwide publications in cancer publications were conducted in African populations. It has been found that most of the last and first authors of these publications and approximately less than 50% were from North African countries. However, 12,65 of the first and last authors of these publications originated from the United States (Rotimi et al., 2021).

Comprehensive perceptions of cancer genomics have been provided by different cancer genomic sequencing studies *via* various consortia, such as the International *Cancer* Genome Consortium (ICGC). However, there is a need to include more racial/ethnic minorities in cancer genomic studies since almost completely studied patients are of European ancestry. This may lead to the identification of novel recurrent mutations that may be ancestral related. This lack of diversity in cancer samples may be due to limited specimens of minority populations coming from academic research centers (Huang, 2018).

Today, establishing a workforce to produce more scientists in Africa focusing on cancer genomics research with a clear roadmap and various stakeholders is needed. Thus, creating a healthy and suitable environment for African researchers in the different research universities across Africa by providing funding for research projects, various research scholarships, rewarding and institutionalizing mentoring of early career researchers, and fostering research experiences is fundamental to limit and prevent ‘brain drain’ from Africa. (Odedina and Rotimi, 2021).

In general, if scientific organizations provide directions, diverse collaborations and well-prepared African scientists, the CGR enterprise can take place in Africa to overcome the lack of African genomic data and biosamples and lead to an outstanding African-led research enterprise (Odedina and Rotimi, 2021).

## Conclusion

There are few studies on the omics of PCa in African populations that concern only genomics (WGS and GWAS), proteomics, and metagenomics of this disease. GWASs revealed 94 PCa-associated variants in these populations. However, more advanced genomic data are mainly needed to discover all PCa-associated variants and to identify all mutation types and chromosomal rearrangements that would be instrumental in stratifying PCa in the African populations, hence providing a population-specific diagnosis and treatment. Promising advances in next-generation sequencing (NGS) tools aside from the decreasing costs of DNA sequencing will allow researchers in Africa to conduct more omics studies on PCa. Collaborations between African research groups and different African and international organizations and consortia may efficiently facilitate and correctly conduct more genomic studies, which will lead to an accumulation of African data that may explain the etiology, clinical heterogeneity, and racial disparities of this disease at the omics level. In Africa, new genomic research resources have been created by recent African consortia and projects such as the Human Heredity and Health in Africa (H3Africa) that help to face challenges related to funding, lack of expertise, and expansion infrastructure for genomic medicine research in general [108].

PCa genomic research on the African population is essential to identify their unique associated variants and investigate their functionality to fully understand the underlying mechanisms of this type of cancer and identify novel targets for diagnosis, treatment, and prognosis by analyzing the genetic profile of each patient and implementing the concept of precision medicine cancer further, which will not leave Africa behind. For instance, establishing an “African Prostate *Cancer* Genomics Consortium” under the umbrella of the International Prostate Genome *Cancer* Consortium becomes a priority to promote more projects and reinforce research activities for PCa omics in the African continent for the benefit for all.
