# Supplementary material for: Review of prostate cancer genomic studies in Africa
Source: Front Genet. 2022 Oct 11;13:911101. doi: 10.3389/fgene.2022.911101 (PMC9593051; doi:10.3389/fgene.2022.911101)
Supplement: Supplementary file 1 [file Table1.DOCX]

**Supplementary material**

**Table S1.** Significant variants associated with prostate cancer(94) identified in Afro-native populations.

| **SNP ID** | **Gene** | **Allele/risk allele** | **Country** | **Ref** |
| --- | --- | --- | --- | --- |
| rs7918885 | - | T \| G | Ghana | [72] |
| rs10905371 | - | A \| G | Ghana | [72] |
| rs7896254 | - | G \| A | Ghana | [72] |
| rs10905374 | - | G \| A | Ghana | [72] |
| rs7096374 | - | C \| T | Ghana | [72] |
| rs61749035 | - | G \| T | Ghana | [72] |
| rs114246623 | - | G \| A | Ghana | [72] |
| rs147739031 | - | A \| G | Ghana | [72] |
| rs115850745 | - | A \| G | Ghana | [72] |
| rs10961884 | - | T \| C | Ghana | [72] |
| rs115899206 | - | A \| G | Ghana | [72] |
| rs12477565 | - | G \| T | Ghana | [72] |
| rs12477565 | - | T \| C | Ghana | [72] |
| rs7090925 | - | A \| G | Ghana | [72] |
| rs17097185 | - | C \| G | Ghana | [72] |
| rs114799364 | - | C \| T | Ghana | [72] |
| rs4151685 | - | A \| C | Ghana | [72] |
| rs34575154 | - | A \| G | Ghana | [72] |
| rs370971 | - | G \| A | Ghana | [72] |
| rs 6878145 | - | A \| G | Ghana | [72] |
| rs2993385 | - | T \| C | Ghana | [72] |
| rs1329536 | - | C \| T | Ghana | [72] |
| rs116776862 | - | G \| A | Ghana | [72] |
| rs13432692 | - | C \| T | Ghana | [72] |
| rs75404762 | - | T \| C | Ghana | [72] |
| rs73146440 | - | A \| G | Ghana | [72] |
| rs285198 | - | G \| A | Ghana | [72] |
| rs12057381 | - | G \| A | Ghana | [72] |
| rs116679801 | - | C \| G | Ghana | [72] |
| rs2056150 | - | G \| A | Ghana | [72] |
| rs7706544 | - | C \| T | Ghana | [72] |
| rs15338764 | - | G \| A | Ghana | [72] |
| rs6889768 | - | T \| G | Ghana | [72] |
| rs6008813 | - | G \| A | Ghana | [72] |
| kgp22385671 | - | A \| G | Ghana | [72] |
| rsll3425597 | - | G \| C | Ghana | [72] |
| rsl2537079 | - | T \| G | Ghana | [72] |
| rs6880234 | - | C \| G | Ghana | [72] |
| rsl2477565 | - | G \| T | Ghana | [72] |
| rsl 7097185 | - | C \| G | Ghana | [72] |
| rsl7119623 | - | T \| C | Ghana | [72] |
| rs6878145 | - | A \| G | Ghana | [72] |
| rs7715021 | - | C \| G | Ghana | [72] |
| rs985081 | - | T \| C | Ghana | [72] |
| rs2185710 | - | A \| G | Ghana | [72] |
| rs66504230 | - | T \| C | Ghana | [72] |
| rsl 14918764 | - | C \| T | Ghana | [72] |
| rs73043340 | - | C \| T | Ghana | [72] |
| rs62477096 | - | A \| G | Ghana | [72] |
| rs6965492 | - | G \| T | Ghana | [72] |
| rs72725854 | - | T | Uganda | [73] |
| rs114798100 | - | G | Uganda | [73] |
| rs72725879 | - | T | Uganda | [73] |
| rs16901979 | - | A | Uganda | [73] |
| rs6983561 | - | C | Uganda | [73] |
| rs111906932 | - | A | Uganda | [73] |
| rs1512268 | - | T | Uganda | [73] |
| rs3096702 | - | A | Uganda | [73] |
| rs11568818 | - | T | Uganda | [73] |
| rs10086908 | - | T | Uganda | [73] |
| rs684232 | - | C | Uganda | [73] |
| rs7463326 | - | G | Uganda | [73] |
| rs12549761 | - | C | Uganda | [73] |
| rs7153648 | - | C | Uganda | [73] |
| rs75823044 | - | T | Uganda | [73] |
| rs1218582 | - | G | Uganda | [73] |
| rs1053005 | 72AT3 | T/C | Tunisia | [102] |
| rs8074524 | STAT3 | C/T | Tunisia | [102] |
| rs3809758 | STAT3 | C/T | Tunisia | [102] |
| rs7045455 | SMARCA2 | T/C | Tunisia | [102] |
| rs12686439 | SMARCA2 | G/A | Tunisia | [102] |
| rs10810919 | SMARCA2 | T/C | Tunisia | [102] |
| rs10963533 | SMARCA2 | T/C | Tunisia | [102] |
| rs10963540 | SMARCA2 | G/A | Tunisia | [102] |
| rs12601982 | STAT5A | A/G | Tunisia | [102] |
| rs8078731 | STAT3 | A/T | Tunisia | [102] |
| rs5750627 | LOC646851 | C/T | Tunisia | [102] |
| rs6001173 | LOC646851 | C/T | Tunisia | [102] |
| rs138702 | SUN2 | T/A | Tunisia | [102] |
| rs138712 | SUN2 | A/G | Tunisia | [102] |
| rs9364554 | *SLC22A3* | - | South Africa | [74] |
| rs10486567 | *JAZF1* | - | South Africa | [74] |
| rs6465657 | *LMTK2* | - | South Africa | [74] |
| rs7008482 | *NSMCE2* | - | South Africa | [74] |
| rs6983561 | *PRNCR1* | - | South Africa | [74] |
| rs6983267 | *POU5F1P1* | - | South Africa | [74] |
| rs4242382 | *POU5F1P1* | - | South Africa | [74] |
| rs10993994 | *MSMB* | - | South Africa | [74] |
| rs4962416 | *CTBP2* | - | South Africa | [74] |
| rs7931342 | *MYEOV* | - | South Africa | [74] |
| rs4430796 | *HNF1B* | - | South Africa | [74] |
| rs1859962 | *CASC17* | - | South Africa | [74] |
| rs2735839 | *KLK3* | - | South Africa | [74] |
